# The geographical and seasonal mosaic in a plant-herbivore interaction: patterns of defences and herbivory by a specialist and a non-specialist

**DOI:** 10.1038/s41598-019-51528-8

**Published:** 2019-10-23

**Authors:** Diomar Verçosa, Rodrigo Cogni, Marcos Nopper Alves, José Roberto Trigo

**Affiliations:** 1 Postgraduate Program in Plant Biology, Institute of Biology, University of Campinas - UNICAMP, Campinas, SP Brazil; 2 Laboratory of Plant Tissue Culture, Department of Agrotechnology, Center for Biological and Agricultural Chemistry, UNICAMP, Campinas, SP Brazil; 3 Laboratory of Chemical Ecology, Department of Animal Biology, Institute of Biology, University of Campinas - UNICAMP, 13083-862 Campinas, SP Brazil; 4 Department of Ecology, University of São Paulo, São Paulo, 05508-900 Brazil

**Keywords:** Coevolution, Secondary metabolism, Entomology, Evolutionary ecology, Tropical ecology

## Abstract

In order to evaluate the geographic mosaic theory of coevolution, it is crucial to investigate geographical variation on the outcome of ecological interactions and the functional traits which dictate these outcomes. Plant populations are attacked by specialist and non-specialist herbivores and may have different types of chemical and biotic defences. We investigated geographical and seasonal variation in the interaction between the plant *Crotalaria pallida* and its two major herbivores (the specialist *Utetheisa ornatrix* and the non-specialist *Etiella zinckenella*). We first showed that attack by the two herbivores and a chemical and a biotic defence vary greatly in time and space. Second, we performed a common garden experiment that revealed genetic variation among populations in herbivore resistance and a chemical defence, but no genetic variation in a biotic defence. Third, we sampled 20 populations on a much larger geographical scale and showed great variation in attack rates by the two herbivores and a chemical defence. Finally, we showed that herbivory is not correlated with a chemical defence in the 20 field populations. Our study shows that to understand the evolution of ecological interactions it is crucial to investigate how the outcome of the interaction and the important species traits vary geographically and seasonally.

## Introduction

Coevolution, the reciprocal evolutionary change in interacting species driven by natural selection, is a central theme in studies of evolution of species interactions^1,2^. Since 1964, when Ehrlich and Raven popularized the term^3^, coevolution has been used in reference to different patterns and processes occurring at different scales of biological organization. Thompson^1,4,5^ introduced the geographic mosaic theory of coevolution and argued for the need of studies of geographical variation in ecological interactions. Therefore, to comprehend the evolution of ecological interactions it is essential to investigate how the outcome of the interaction and the important species traits varies geographically.

One of the best studied type of ecological interaction is the interaction between plants and herbivorous insects^6^. Herbivorous insects impose an important selective pressure on plants by consuming a large part of plant biomass in natural communities^7,8^. To avoid or reduce the loss of biomass, plants evolved a diverse array of defences such as the production of compounds toxic to herbivores and/or deterrents and physical barrier as leaf hardness, thorn and trichomes (all types of direct defenses, as they act directly on the herbivore)^9–11^. In response to plant chemical defences, herbivorous insects evolved a wide range of physiological, morphological and behavioural adaptations^8^, including avoidance, excretion, detoxification or sequestration of the toxin^12,13^. Over evolutionary time, herbivores may became specialized in plants with similar chemical compounds and evolve adaptations to sequester and use them as predator defence^14,15^. For example, pyrrolizidine alkaloids (PAs) are compounds toxic or deterrent to several generalist polyphagous herbivores^15–17^. However, they are sequestered by specialist herbivores, stored in the tissues in a metabolic safe form^15,18^ and utilized against predators^15,19^.

Another strategy of plant defence is the indirect or biotic defences^10,11^, such as the attraction of natural enemies of herbivores (e.g. predatory ants and wasps), attracted by nutritive compounds (sugars and amino acids) produced in extrafloral nectaries (EFNs)^20^. EFNs are secretory structures not involved in pollination found in different plant structures^21^. In many systems ants are the main visitors of EFNs. The visiting ants generally attack the herbivores and consume or drive them off the plants, decreasing the damage and, consequently, improving plant fitness^20,22^. However, the benefits of these associations are dependent of the abundance and aggressiveness of the ants present^20,23,24^. In some systems, predatory wasps can also visit the EFNs and have a positive effect on plant fitness^25,26^.

Herbivory may vary spatially and temporally as a result of the quality of plant in terms of defensive compounds^6^ and indirect defences^27^. Herbivores can be present in high density in some locality and absent in others. The presence or absence of herbivores or other organisms may affect the result of the interactions. For example, spatial variation in the presence of natural enemies (birds and parasitoid wasps) determines the size of galls induced by a fly^28^. Some studies showed that spatial variation in abundance of a specialist and a generalist herbivore also affect the main chemical defence of a plant, in the absence of the specialist herbivore, selection favoured the increase in the concentration of the defence, while in the presence of the specialist the trait remained neutral^29,30^. In relation to indirect defences, Sendoya & Oliveira^27^ found a negative relation between the infestation of plants by caterpillars and the presence of visiting ants, and this relation varied geographically depending on the abundance and composition of the ant species. Nogueira, *et al*.^31^ verified that the biggest spatial difference in the outcome of the interactions was due to the difference in the species and behaviour of the ants visiting the EFNs.

In this study we investigate geographical and seasonal variation in the outcome of a plant-herbivore interaction. Legumes of genus *Crotalaria* (Papilionoideae: Crotalarieae) are an ideal system to study this problem because they contain chemical defences such as pyrrolizidine alkaloids (PAs) and biotic defences such as EFNs, and are attacked by the specialist moth *Utetheisa ornatrix* (Erebidae: Arctiinae) and the non-specialist *Etiella zinckenella* (Pyralidae). Previous studies in this system have focused on the interaction between the specialist herbivore *U. ornatrix* and its main host plant *C. pallida*. There is geographical variation in the interaction between *C. pallida* and *U. ornatrix* and local adaptation by the herbivore depends on the geographical scale^32,33^. The EFNs in *C. pallida* represent an effective mechanism of protection against the attack of the herbivore specialist *U. ornatrix*^34^, but *U. ornatrix* larvae feeds mainly inside the pods where they are protected from ants and are able to increase the sequestration of PAs, as seeds have higher PA concentration than leaves^35^. The PA concentration of the host species that the larvae feed on affects the level of protection of *U. ornatrix* adults against predators; feeding on host plants with the highest concentration of PAs provide the most efficient protection against the spider *Nephila clavipes*^19^. Recent studies in this system showed that the specialist herbivore *U. ornatrix* is not affected negatively by the concentration of PAs, and the sequestration of these compounds does not have a fitness cost^36^. Additionally, the larvae prefer diets with high concentration of alkaloids^37^. These features make *U. ornatrix* a potential agent of selection to lower the levels of chemical defensive compounds in population of its host plant. However, the presence of other herbivores, with different level of specialization, can influence the results of the interactions^29,38^. The importance of the non-specialist herbivore *E. zinckenella* has not been explored in this system^39,40^. Plants faced with different herbivores with different levels of specialization may differ in defence strategies. It is expected that the specialist *U. ornatrix* would act as selection agent that decreases the concentration of PAs, while the non-specialist *E. zinckenella* would act as a selection agent that increases the concentration of PAs.

We studied geographical and seasonal variation in the interaction between *C. pallida* and its two major herbivores (the specialist *Utetheisa ornatrix* and the non-specialist *Etiella zinckenella*). We first showed that attack by the specialist and the non-specialist herbivore, as well as a chemical defence (PAs) and a biotic defence (EFNs), vary greatly in time and space. Second, we performed a common garden experiment that revealed genetic variation among populations in herbivore susceptibility and the chemical defence, but no genetic variation in the biotic defence. Third, we expand our sampling to 20 populations on a much larger geographical scale and showed great variation in attack rates by the specialist and the non-specialist herbivore as well as the chemical defence. Finally, we showed that herbivory by the specialist and the non-specialist is not correlated with the chemical defence in the 20 field populations.

## Material and Methods

### Study organisms

**Figure 1:**
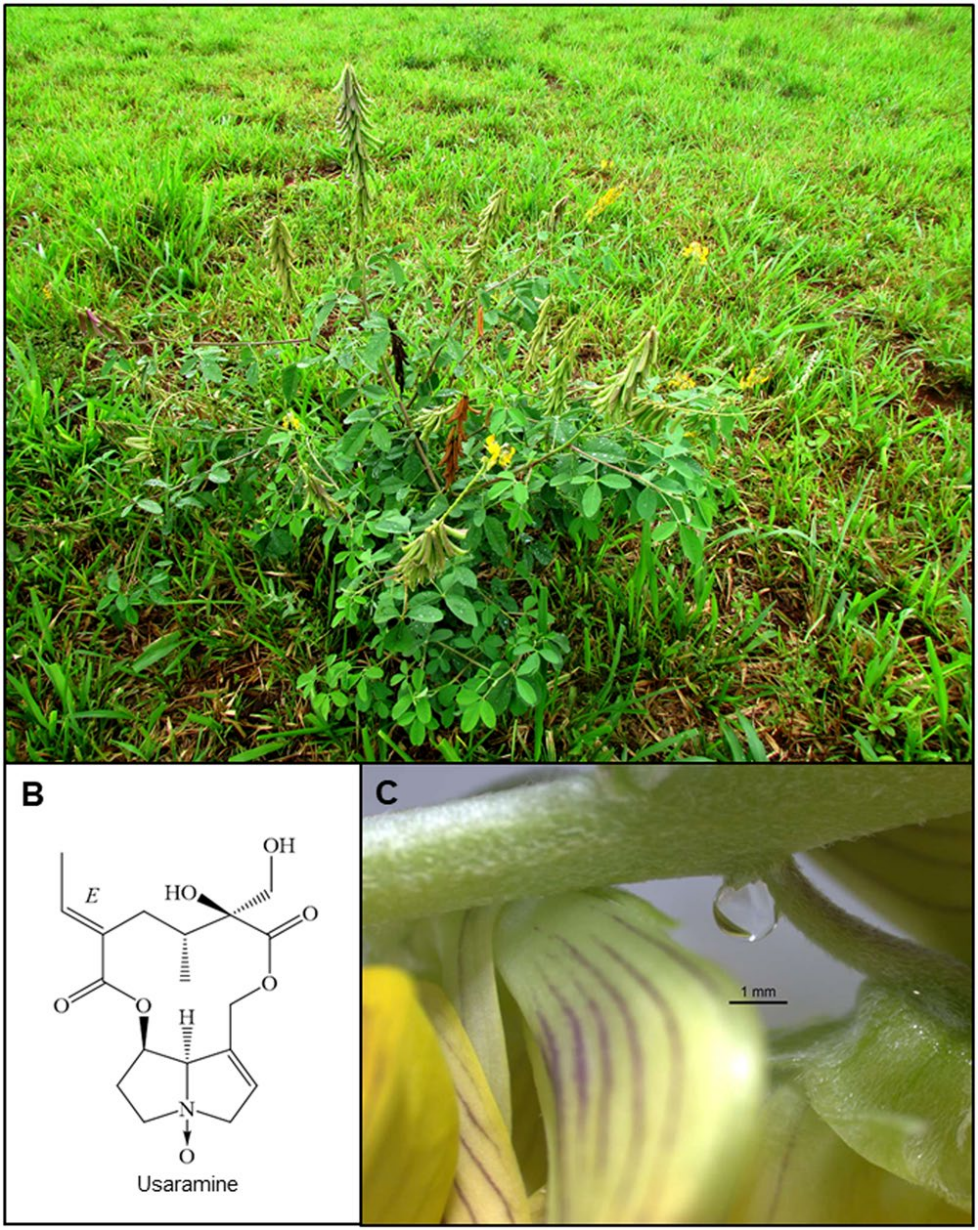
Individual of *Crotalaria pallida*, with flowers and pods (**A**), chemical structure of the main pyrrolizidine alkaloid in the form *N*-oxide, usaramine (**B**), and extrafloral nectaries located at pedicel base with a drop of nectar secreted (**C**).

The genus *Crotalaria* (Fabaceae: Papilionoideae: Crotalarieae) comprises approximately 702 species, occurring mainly in the tropics and subtropics^41^. In Brazil there are 42 species, with 31 native and 11 introduced (Flores 2004). *Crotalaria pallida* has a wide distribution, occurring almost everywhere in disturbed places, such as in vacant lots, roadsides and pastures^42^. *Crotalaria pallida* is a 0.6–1.5 m tall herbaceous annual (Fig. 1A) and has Pantropical distribution; its biogeographic origin is obscured by its wide distribution and rapid naturalization^43^. *Crotalaria pallida* is a species with high invasive potential in the Brazilian biomes^44^. This species has chemical defences based on PAs, presenting as major alkaloid the usaramine^45^ (Fig. 1B); the PAs are always present in the N-oxide form^19^. *Crotalaria pallida* has also indirect defences based on the attraction of predators such as ants and wasps to their EFNs^32,33,46^ (Fig. 1C), which are located at the pedicel base (Edna Scremin Dias pers. com.), as described by Díaz-Castelazo *et al*.^47^ in *C. incana*.

**Figure 2:**
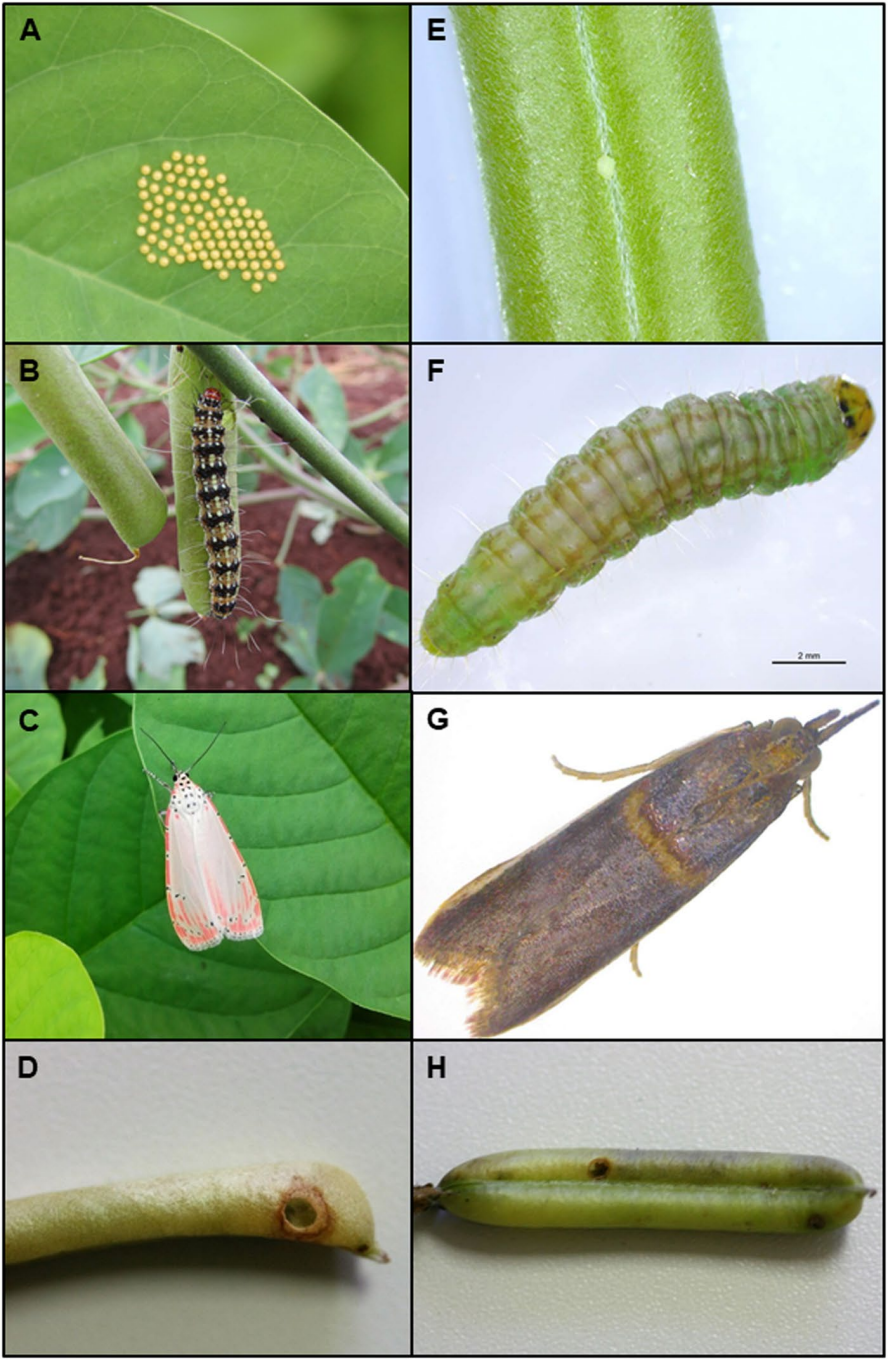
Grouped eggs (**A**), larva (**B**), adult (**C**) and perforated pod by larva of *Utetheisa ornatrix* to enter the pod (**D**); egg (**E**), larva (**F**), adult (**G**) and perforated pod by larva of *Etiella zinckenella* to leave the pod (**H**) of *Crotalaria pallida*.

*Utetheisa ornatrix* is a neotropical moth which flies diurnally and larvae feed on different *Crotalaria* species^19,48–50^. The females lay their eggs grouped in the leaves (Fig. 2A). As larvae hatch, they feed on leaves in the first instars and then perforate the pods to feed on unripe seeds^32^; even first instar larvae can perforate new pods, if available (J.R. Trigo, pers. com.). A single larva (Fig. 2B) consumes, on average, three pods of *C. pallida* until they reach adult stage (Fig. 2C) (D. Verçosa pers. com.). During the larval stage, the main natural enemies are ants and wasps, which are attracted by EFNs^26,33^. Both seeds and leaves have PAs, which are sequestered by larvae and used as defensive compounds and precursors of sex pheromones^19,48^. Larvae are also attacked by parasitoids such as tachinid flies^51^, chalcidid, ichneumonid and brachonid wasps^52^ (J.R. Trigo pers. com.).

*Etiella zinckenella* (Fig. 2E–G) is a cosmopolitan moth which flies nocturnally and larvae feed on several genus of Fabaceae^39^. In Brazil, it is known as one of the main pest responsible for the damage in the production of beans (*Phaseolus vulgaris*) of Rio Grande do Sul, due to the damage caused to seeds^53^. *E. zinckenella* was reported to feed in five species of *Crotalaria*: *C. pallida*, *C. incana*, *C. micans*, *C. zanzibarica* (=*C. trichotoma*), *C. vitelina*^40,54^ (JR Trigo, pers. com.). *C. pallida* is considered one of the most susceptible species to the attack^54^. *E. zinckenella’s* main natural enemies are parasitoid brachonid and chalcidid wasps^54,55^.

### Field sites

The first part of this study was conducted from January 2012 to January 2013 in three localities in the state of São Paulo, southeastern region of Brazil, about 70 km apart; Iperó (23°24'41,8″S, 47°42'32,1″W), Martinho Prado (22°17'06,2″S, 47°08'30,1″W) and Campinas-Village (22°44'43,3″S, 47°03'44,5″W). The second part was conducted from January to May of 2014 in twenty locations in the Southeast, South and Center-West of Brazil, in the states of Paraná, Mato Grosso do Sul, São Paulo and Rio de Janeiro (Fig. S1, Table S1). The localities were chosen according to the occurrence of *C. pallida*, and they have the same physiognomy, characterized by the predominance of grasses of the genus *Brachiaria* (Poaceae).

### Herbivory

In all samples, the numbers of attacked pods for each herbivore were counted on three branches of each plant (*n* = 10 per locality) as an estimate of herbivory. We considered pods attacked by *U. ornatrix*, those that contained larvae of the herbivore in their interior, as well as pods without larvae but with preyed seeds and perforation made from outside to inside of the pod (Fig. 2D). For *E. zinckenella* attacks, we considered the pods which contained larvae inside them and pods without larvae but with preyed seeds and perforation made from inside to the outside of the pod (Fig. 2H). We separated the percentage of attacked pods in populations into three categories: plants attacked by *U. ornatrix*; plants attacked by *E. zinckenella* and plants attacked by both herbivores simultaneously (hereafter this variable will be referred to as herbivore type).

### Chemical defences

To assess whether the PA concentration in unripe seeds of plants attacked by herbivores varies among the populations of *C. pallida*, we homogenized approximately 100 mg of freeze-dried unripe seeds from each sampled plant. In each population, 400 pods of 10 plants were collected on average. Homogenization for 1 minute was done in a test tube (1.3 cm diameter, 10 cm height) with sea sand (J.T. Baker) and 2 ml of ethanol. The homogenate was centrifuged for 5 minutes at 805 × g, the supernatant was removed, and the residue of the seeds was extracted more two times, as described above. After, we combined the three supernatants, and ethanol was added to bring the volume to 10 ml. For quantification, we removed a 1 ml aliquot of the 10 ml solution and quantified using the modified colorimetric method of Mattocks^56^ as described in Martins *et al*.^19^. We performed the reading of the absorbance of the samples in triplicate, at wavelength 561 nm, using a SP-22 digital spectrophotometer, Biospectro brand (reading range 325–1000 nm) with resolution of ±0.078 A. We quantified the PAs by converting the mean values of absorbance in μg, based on a calibration curve, prepared with the PA monocrotaline alkaloid standard (absorbance = 0.0389x μg monocrotaline, r^2^ = 0.980). We assume that the molar extinction coefficient does not vary between PAs. PA concentration is shown in μg/g of seeds dry weight.

### Biotic defences

The effect of predators attracted by EFNs was evaluated in a bait removal bioassay, in which we used as a model of prey termites, which are frequently used in this type of bioassay (e.g. see Guimarães *et al*.^33^). The termites were collected in an area near to a forest fragment at the University of Campinas in Campinas city, Brazil (22°49′15.38″S, 47°4′8.87″W). We applied this bioassay in the same plants (n = 10), where we sampled the unripe pods in the three populations of *C. pallida*, in the three initial periods. One day before sampling the unripe pods, we marked four branches with unripe pods on each plant. In separate branches of each plant we applied the following treatments: (1) without ants and without wasps [A − W−], (2) with ants and without wasps [A + W−], (3) without ants and with wasps [A − W+], and (4) with ants and with wasps [A + W+]. In each branch, we glued a termite on the first pod from apex using cyanoacrylate based glue (Super Bonder®, Henkel Brazil). Before applying the treatment, we removed ants and other predators that were in the branches. To exclude the ants, we apply Tanglefoot® resin at the base of the branch. To exclude wasps, the branch was wrapped with a tulle bag (50 cm long, 35 cm wide, and 2 mm mesh width). With the exclusion of flying predators, we exclude ants that cannot walk through the mesh of the tulle. So, to exclude wasps and not exclude ants, we put a plastic tube (7.0 cm long, 0.8 cm inner diameter), connecting the outside with the inside of the tulle bag. After 24 hours, we recorded if the termites (hereafter baits) had been removed. We also collected in each locality ants visiting EFNs to characterize the ant community of the place and to analyse if there is relation with the percentage of baits removed.

### Geographic and temporal sampling

To investigate the geographic and temporal variation of the interaction between *C. pallida* and its herbivores, we sampled three populations (Iperó, Martinho Prado and Village) for one year. In this period, we performed eight samplings per population 45 days apart. In each population, we conducted a non-systematic sampling of 10 plants and three branches with unripe pods per plant.

At each sampling, we checked if there were plants in the place and if they had branches with flowers, unripe or ripe pods. These data were used as the basis for the statistical analysis of the following parameters: percentage of attacked pods, concentration of PAs and activity of natural enemies.

### Common garden experiment

To test whether variation observed among populations in susceptibility to herbivores, PA concentrations, and attraction of natural enemies was caused by environment or genetic variation among populations, we performed a common garden experiment in the Chemical, Biological and Agricultural Pluridisciplinary Research Center (CPQBA), UNICAMP. This method consists of cultivating plants of different populations under the same environmental conditions^57^. This type of experiment indicates that if, under the same environmental conditions, differences occur between the measured characters, they should be due to genetic differences between the original populations.

For the cultivation of the plants, seeds collected in April of 2013 from three populations (Iperó, Martinho Prado and Village) were sown in trays containing substrate Gioplanta^®^ mixed with fertilizer Osmocote^®^ (N:P:K = 19:6:10) at a concentration of 4 g/Kg. The source seeds were collected from *ca*. 10 individual plants per population and the seed from different individuals were randomly mixed before they were sown. After germination, we transplanted the seedlings to tubules (5 cm diameter, 15 cm high), with the same substrate, and kept them for three months in a greenhouse, with automatic irrigation system by micro sprinkler, without control of temperature. After this period, we planted the seedlings in the experimental field of the CPQBA.

We randomly planted 33 seedlings from each population, with spacing between individuals of 1.5 m. We performed two samplings, one in January and another in April 2014. From each sampled plant, we randomly collected three branches with unripe pods. The percentage of attacked pods and the PA concentration were measured as described above, and the biotic defences also was measured as described above, with the following modifications: the amount of baits used in each treatment was five, instead of one.

We also measured the height of the individuals of the three populations, since differentiation in the height of the plants within the common garden experiment may represent an indication of genetic differences between the original populations, and plant size may affect herbivory rates. We did not find significant difference among populations in height in January (One-way ANOVA, F_2,80_ = 2.41, P = 0.097) and April (F_2,25_ = 0.63, P = 0.541).

### Sampling 20 populations over a very large geographical scale

To investigate if there was a correlation between chemical defences of *C. pallida* and its herbivores in a very large geographic scale, in January 2014, we collected samples from twenty populations of *C. pallida* located in the regions of Paraná, Mato Grosso do Sul, São Paulo and Rio de Janeiro, with the shortest distance between the populations being 80 km and the largest 1416 km (Fig. S1, Table S1). We performed a non-systematic sampling of 20 plants in each population. From each plant we collected three branches with unripe pods, in which we measured the percentage of attacked pods by each type of herbivore and used the intact seeds to extract the PAs. In May of 2014, we carried out the collections in the same localities, using the same sampling method, except in Bataguassu, Corumbá, Itanhaém and Miranda, where it was not possible to find plants. We sampled in January and May because our results of the local scale study showed a higher incidence of non-specialist herbivore in December-January and higher incidence of specialist herbivore in April-May.

We used another method of extraction for the unripe seeds samples from the 20 populations, due to the distance from the Laboratory of Ecology, Unicamp, which made it impossible for the samples to be taken for lyophilization. Therefore, we packed the unripe seeds of each sampled plant in glass vials (volume 15 mL) with EtOH. To conduct PA extraction, we first remove the alcoholic solution and transfer it to a 50 mL Falcon tube with a screw cap. We transferred the seeds to a test tube (2.5 cm in diameter × 12.6 cm in height) and added 10 mL of EtOH. Then, homogenized for 30 seconds in the homogenizer (Marconi, model: MA 102). We washed the homogenizer stem and the tube with 10 mL of EtOH and transferred the extract to a Falcon tube for centrifugation for 15 minutes at 10416 × g in a Bekcman Coulter Allegra X-30R Centrifuge. We removed the supernatant with the aid of a pipette. We added 20 mL of EtOH to the Falcon tube, shake for 30 seconds on the tube shaker (Cetomart), centrifuged again and repeated this step again. We assembled the three supernatants into a 100 mL volumetric flask and completed the volume to that extent with EtOH. We removed a 3 mL aliquot of the 100 mL solution for quantification by colorimetric method as described above.

These data were used to test the hypothesis that populations with higher incidence of the specialist *U. ornatrix* would present a lower PA concentration, where those with a higher relative incidence of the non-specialist *E. zinckenella* would present a higher concentration of these alkaloids. In populations where both herbivores had a similar incidence, we would expect an intermediate concentration.

### Statistical analyses

The details of the statistical analyses performed to analyse each of the data sets collected are presented in the supplemental material (Supplemental Material 1).

## Results

### Attack by a specialist and a non-specialist herbivore varies in time and space

We observed that *C. pallida* present fruits during most of its life cycle, but with different intensities. Flowering occurred predominantly in January and February, branches with unripe pods were presented in March to June, in July the pods dried up and the plants died, new individuals began to emerge in November. This period of fruiting was variable among the populations sampled. For example, the population of Martinho Prado remained for a longer time with unripe pods compared to the populations of Iperó and Village. On the other hand, it did not present new individuals in November and December, when new individuals began to emerge in the other two populations.

**Figure 3:**
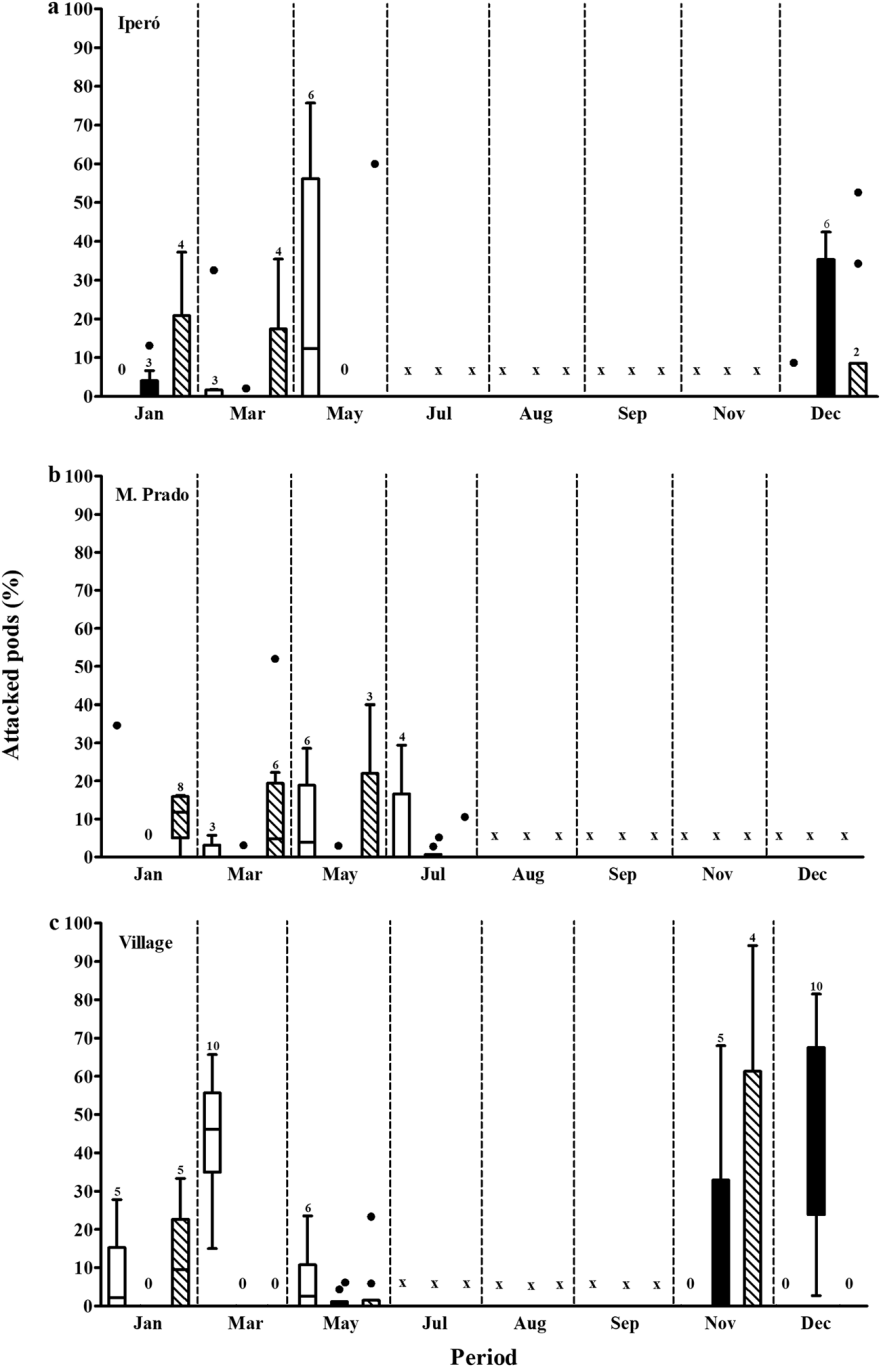
Percentage of attacked pods by *Utetheisa ornatrix* (white) and *Etiella zinckenella* (black) or both (dashed) in the population of *Crotalaria pallida* of Iperó (**a**), Martinho Prado (**b**) and Village (**c**) during the year. “0” indicates that there was no herbivory on the plants in the population and period. “x” indicates that plants were not found in the period. Data are present as median and interquartile range. The dots represent outliers. Values above bars indicate the number of sampled plants.

The percentage of attacked pods by *U. ornatrix* and *E. zinckenella* differed significantly among populations, between the herbivores, and with all the interaction among the factors (Table S2). On average the Village population showed 12,3% higher percentage of attacked pods than the population of Iperó, and 19,5% higher than the population of Martinho Prado (Fig. 3). The percentage of attacked pods by *U. ornatrix* and both herbivores simultaneously were similar and *E. zinckenella* showed lower percentage of attacked pods overall. The percentage of attacked pods by *U. ornatrix* was higher in Iperó, in the period May/Jun and in the Village in the period Mar/Apr (Fig. 3). In addition, during the year, the three populations did not show unripe pods in the periods of Aug/Sep and Sep/Oct, therefore, no herbivore was sampled. In the periods of Nov/Dec and Dec/Jan, the populations of Iperó and Village were mainly attacked by *E. zinckenella* (Fig. 3). We found a higher percentage of attacked pods by *U. ornatrix* in the periods of Jan/Feb and Mar/Apr, and by *E. zinckenella* in the periods of Nov/Dec and Dec/Jan (Fig. 3).

### A chemical defence varies in time and space

**Figure 4:**
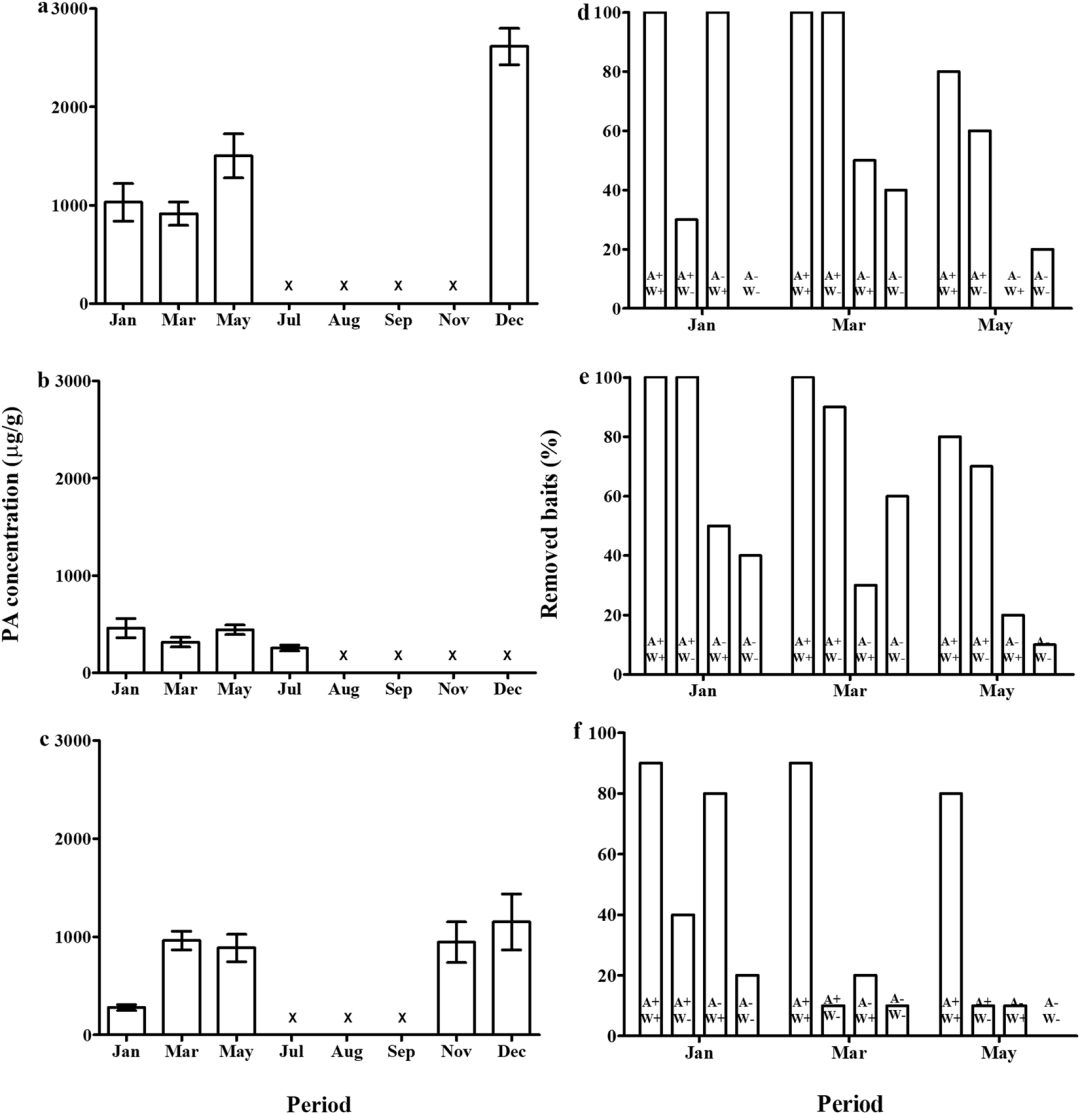
**(a–c)** Pyrrolizidine alkaloids concentration in unripe seeds in three population of *Crotalaria pallida*, along the year in Iperó (**a**), Martinho Prado (**b**), and Village (**c**). “x” indicates that plants were not found in the period. Data are present as mean ± standard error (n = 10). **(d–f)** Percentage of removed baits in three populations of *Crotalaria pallida*, Iperó (**d**), Martinho Prado (**e**) and Village (**f**), in the periods and in four treatments (A + W+: with ants and wasps, A + W−: with ants and without wasps, A − W+: without ants and with wasps, and A − W−: without both predators).

We verified PA concentration of unripe seeds on the same populations and periods where we sampled the percentage of attacked pods. PAs differed significantly among populations (Two-way ANOVA, F_2,80_ = 36.987, *P* < 0.001), periods (F_2,80_ = 10.402, *P* < 0.001) and the interaction between population and period (F_4,80_ = 8.793, *P* < 0.001, Fig. 4). The population of Iperó showed the higher PA concentration, 3x more than the concentration of the population of Martinho Prado (Fig. 4). There was also great variation among periods (Fig. 4).

### A biotic defence varies in time and space

We also investigated, in the same three population, bimonthly from January to June, the efficient of predators attracted by EFNs with an experiment that excluded wasps and ants. We found that the percentage of removed baits varies among the populations depending of the period and treatment (Table S3). In the population of Village there was lower percentage of removed baits compared to Iperó and Martinho Prado (Figs 4, S2a). Among the periods, we verified higher percentage of removed baits in the periods Jan/Feb and Mar/Apr than May/Jun (Figs 4, S2b). In general, the percentage of removed baits was high, considering ants and wasps together, and ants removed more baits compared to wasps (Figs 4, S2c). In the Village population, when the wasps were present there was an additive effect in the percentage of removed baits (Fig. 4). The ant community visiting the EFNs also varied among populations. In the population of Iperó, *Solenopsis* species were predominant, with number of individuals above 20 per plant (Table S4). We also found nests of these ant species at base of the plants. In Martinho Prado, we observed the predominance of ant species of the genus *Pheidole*, which presented intense recruitment behaviour. In contrast, in the population of Village, there was the predominance of large *Camponotus* species with weak recruitment behaviour (Table S4).

### A common garden experiment reveals genetic variation among populations in herbivore resistance and chemical defence, and no variation in a biotic defence

**Figure 5:**
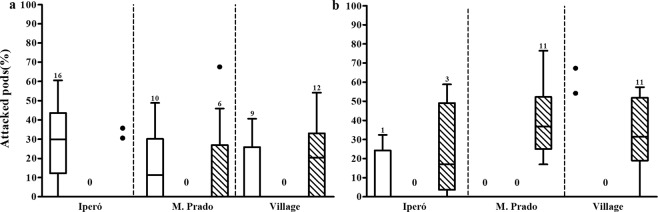
Percentage of attacked pods by *Utetheisa ornatrix* (white), Etiella (black), and both herbivores simultaneously (dashed) in the three populations of *Crotalaria pallida* in January (**a**) and April (**b**) on a common garden experiment. “0” indicates that no herbivory was found in the period. Data are present as median and interquartile range. The dots represent outliers. Values above bars indicate the number of sampled plants.

To test if the differences observed among populations in the field are determined by genetic differentiation among populations, we grew plants of the three populations under the same environmental conditions. First, we looked at the percentage of attacked pods in January and April. In January we did not find significant difference among the populations (Table S5), and most pods were attacked by *U. ornatrix*, followed by attacks by both herbivores simultaneously, and no pods were attacked only by *E. zinckenella* (Fig. 5a). There was a significant interaction between population and herbivore type suggesting some genetic differentiation among populations in traits affecting attack by the different herbivores (Table S5). In April, we did not observe significant difference among the populations in the percentage of attacked pods but found differences among herbivore types (Table S5), and most pods were attacked by both herbivores simultaneously (Fig. 5b).

**Figure 6:**
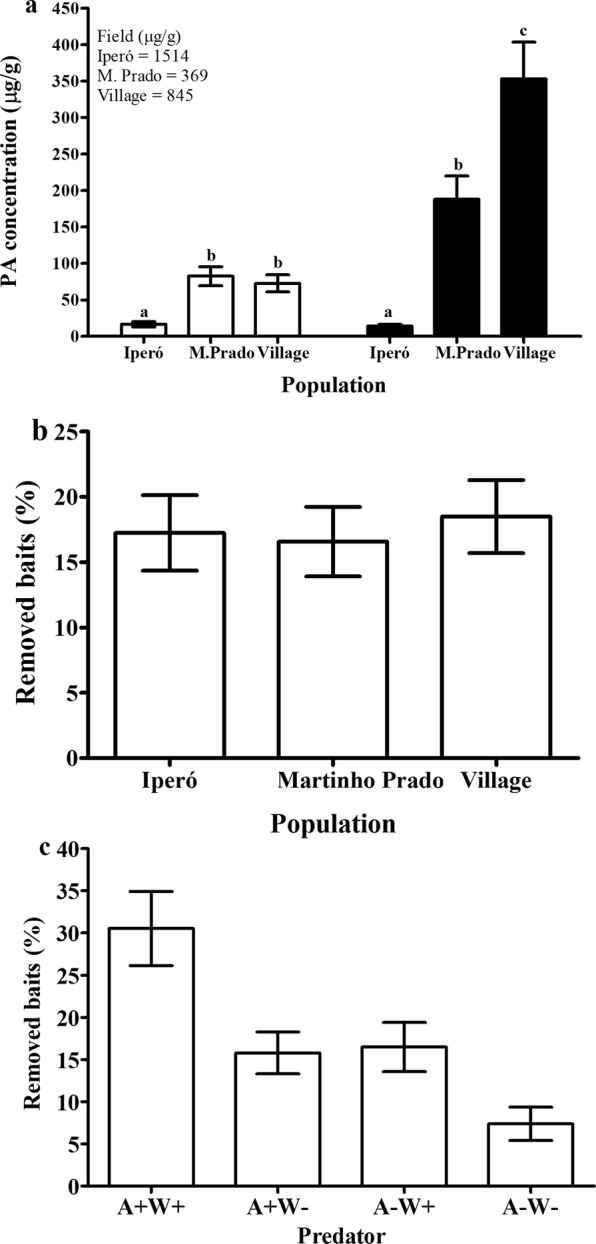
Pyrrolizidine alkaloids concentration in unripe seeds (**a**) in three populations of *Crotalaria pallida* in January (white) and April (black), and percentage of removed baits in three populations (**b**) and in four treatments (A + W+: with ants and wasps, A + W−: with ants and without wasps, A − W+: without ants and with wasps, and A − W−: without both predators) (**c**) on a common garden experiment. Data are present as mean ± standard error (n = 10). Different letters above each bar represent significant differences (P < 0.05). Data at the top of the figure represent the concentration of PAs found in the field.

Second, we looked at PA concentration of unripe seeds. The PA concentration varied significantly among the populations in January (One-way ANOVA, F_2,51_ = 27.884, P < 0.001) and April (F_2,23_ = 40.615, P < 0.001, Fig. 6). The population of Iperó showed much lower PA concentration than Martinho Prado and Village (Tukey test, P < 0.001, Fig. 6). In general, the populations showed lower PA concentration on the common garden compared to concentration observed on the original site (Fig. 6).

Third, we tested the effect of herbivore predators attracted by EFNs with an experiment that excluded wasps and ants. The percentage of removed baits did not differ significantly among the populations (GLM, x^2^ = 0.278, P = 0.870, Fig. 6). There was a significant difference among the treatments (x^2^ = 29.134, P < 0.001); the percentage of removed baits by ants and wasps together was significantly higher than the treatments with only ants, only wasps, and without predators (Tukey test, P = 0.002, 0.001 and <0.001, respectively, Fig. 6).

### Attack by a specialist and a non-specialist herbivore and a chemical defence vary greatly among 20 populations

**Figure 7:**
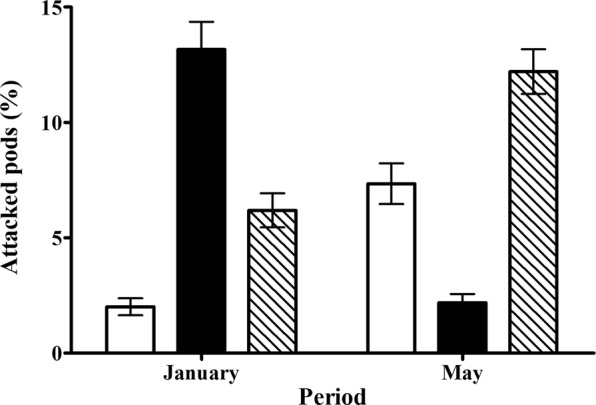
Percentage of attacked pods by *Utetheisa ornatrix* (white) and *Etiella zinckenella* (black) or both herbivores simultaneously (dashed) in the twenty populations of *Crotalaria pallida* sampled in January and May of 2014.

We sample 20 populations over a very large geographical scale spreading up to 1416 km. We found great variation among populations on the percentage of attacked pods by *U. ornatrix* (4.42 ± 12.01), *E. zinckenella* (8.19 ± 18.58) and both herbivores simultaneously (8.81 ± 15.92) (GLM, x^2^ = 355.8, df = 19, P < 0.0001, Fig. S3). Considering the 20 populations in general, we observed that the percentage of attacked pods differed significantly among periods (x^2^ = 12.1, df = 1, P < 0.0001), among herbivore types (x^2^ = 98.3, df = 2, P < 0.0001), and there was a significant interaction among all the factors (Table S6). In January there was a greater percentage of attacked pods by *E. zinckenella*; which decreased from January to May, while the percentage of attacked pods by *U. ornatrix* increased from January to May. The percentage of attacked pods by both herbivores also increased from January to May (Fig. 7). *Etiella zinckenella* was predominant on most populations in January (Fig. S3). Among the 20 sampled populations, 10 of them were dominated by *E. zinckenella*, and in Corumbá and Ibiporã we found attacked pods only by this herbivore (Fig. S3). The highest percentages of attacked pods by this herbivore were found in the populations of Iperó, Bataguassu, Rancharia and Corumbá (Fig. S3). In the population of Corumbá, there were individuals that had about 97% of their pods attacked by *E. zinckenella*. In contrast, the population that showed higher percentage of attacked pods by *U. ornatrix*, Ivinhema, had on average 13% of attacked pods (Fig. S3). In this population there was predation only by *U. ornatrix*. Comparing the percentage of attacked pods between January and May, we found that, in some populations, the percentage of attacked pods by *E. zinckenella* decreased in May and by *U. ornatrix* increased. However, in other populations, the relative proportion of pods attacked by each species did not change from January to May (Fig. S3). In addition, there were populations such as Ibiporã, where we found only *E. zinckenella* in January, but which presented one of the highest percentages of attacked pods by *U. ornatrix* in May (Fig. S3). The greater percentage of attacked pods by both herbivores was found in the population of Martinho Prado and Nova Alvorada do Sul (Fig. S3).

We also found significant variation among populations on PA concentration of unripe seeds (Two-way ANOVA, F_15,59_ = 5.134, *P* < 0.001), and the interaction between population and period (F_15,59_ = 4.202, *P* < 0.001), but no significant differences between periods (F_1,59_ = 1.850, *P* = 0.174). The average PA concentration varied among the populations on the range from 217,79 ± 25,02–1072,06 ± 168,78 µg/g (Fig. S4). The populations of Loanda, Iperó and Igarapava showed significantly the highest PA concentrations compared to the populations of Ivinhema, São Simão and Passos (Tukey test, P < 0.001, Fig. S4) which had the lowest concentrations. Most of the populations such as Corumbá, Itanhaém, Miranda and Duartina showed similar PA concentrations, varying around 520 µg/g (Fig. S4).

### There is no correlation between a chemical defence and herbivory by a specialist and a non-specialist in 20 field populations

**Figure 8:**
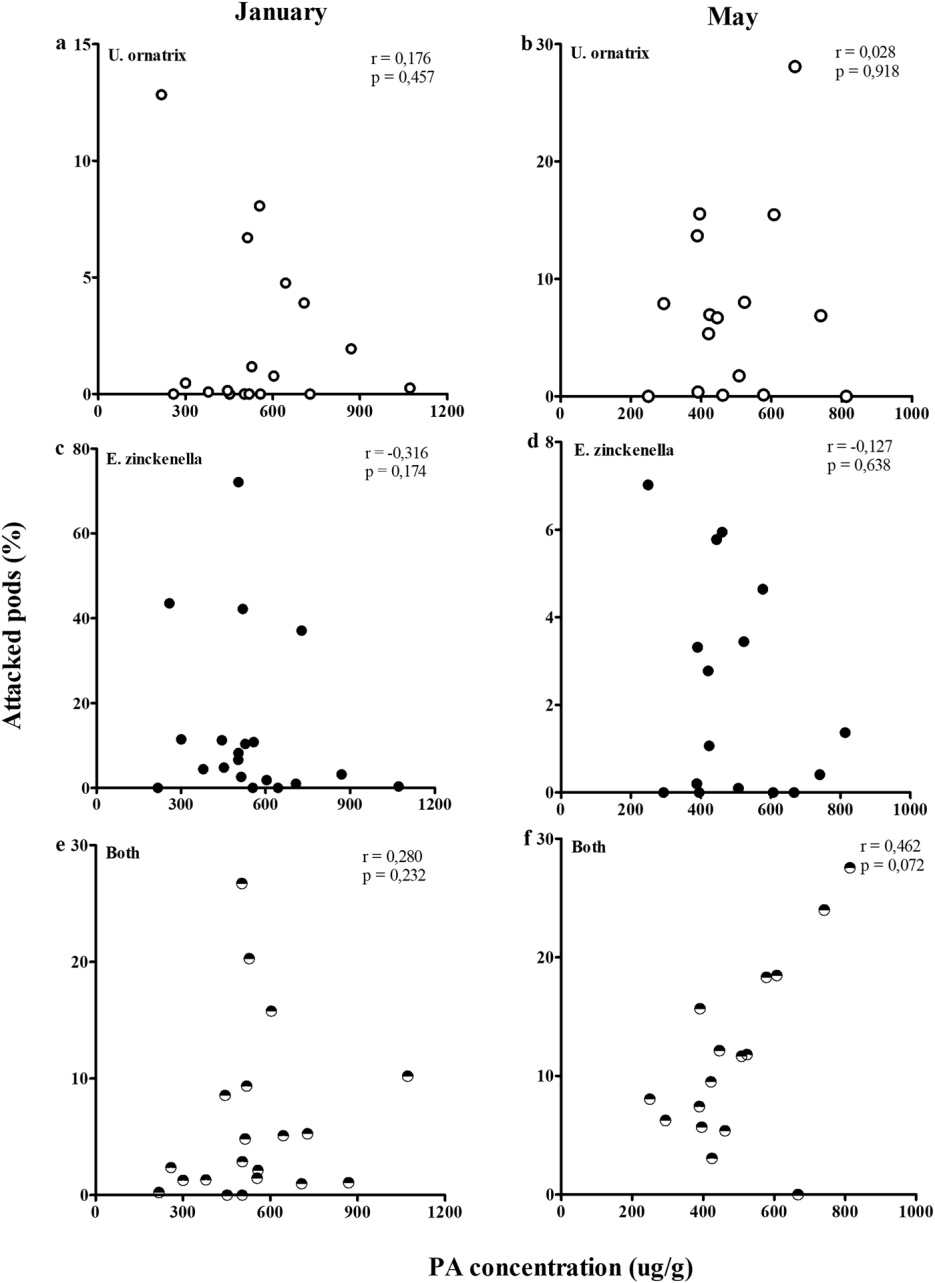
Correlation among the percentage of attacked pods by *Utetheisa ornatrix* (**a**,**b**) and *Etiella zinckenella* (**c**,**d**) or both herbivores simultaneously (**e**,**f**) and the average concentration of pyrrolizidine alkaloids of the unripe seeds of *Crotalaria pallida* of twenty populations in January (left column) and sixteen in May (right column) of 2014.

Given the great variation observed among the 20 populations in chemical defence and herbivory, we tested for correlations between PA concentration and percent of attacked pods by the specialist, the non-specialist and both herbivores simultaneously. We did not find any significant correlation between PA concentration and herbivory rates (Fig. 8; *Utetheisa*: r = 0.176, p = 0.457, *Etiella:* r = −0.316, p = 0.174, both herbivores: r = 0.279; p = 0.232 in January, and *Utetheisa*: r = 0.028, p = 0.918, *Etiella:* r = −0.127, p = 0.638, both herbivores: r = 0.461, p = 0.072 in May).

## Discussion

Our results revealed a wide variation of the percentage of attacked pods among populations and between the herbivores during the year, as well as the importance of *E. zinckenella* as a potential selective agent that have been neglected in previous studies on this system. The percentage of attacked pods by each herbivore type was higher than in previous studies that reported 1.5 to 13% attack by *U. ornatrix* and 2 to 40% for *E. zinckenella*^32,54^. Sampling 20 different populations in a large geographical scale, we found variation of the percentage of attacked pods by *U. ornatrix* from 0 to 15% in January and from 0 to 44% in May, and by *E. zinckenella* from 0 to 72% in January and from 0 to 8% in May. Sampling intensely over the entire year allowed us to reveal that *E. zinckenella* peaks occur in November to January and *U. ornatrix* from March to June. It is important to emphasize that previous studies in this system have focused in the interaction between *Crotalaria* and its specialist herbivore *U. ornatrix*^32,33,35,40^. However, this study revealed that the non-specialist herbivore *E. zinckenella* is responsible for a large part of the percentage of attacked pods, which makes it a potential selective agent that may influence defence levels in populations of *C. pallida*.

The concentration of PAs on unripe seeds of *C. pallida* also varied among the populations and during the year. Some studies demonstrated geographic variation in the level of herbivory and chemical defences^58,59^. For instance, Castells *et al*.^58^ found geographic variation in the concentration of piperidine alkaloids in leaves of *Conium maculatum* (Apiaceae), as well as in the level of herbivory by larvae of the moth *Agonopterix alstroemeriana* (Oecophoridae). Similarly, Castillo *et al*.^59^ verified geographic variation in the concentration of tropane alkaloids (atropine and scopolamine) of *Datura stramonium* (Solanaceae) and in the herbivory by specialist herbivores (the beetle chrysomelidae *Lema daturaphila* and *Epitrix parvula*) and the generalist (grasshopper pyrgomorphidae *Sphenarium purpurascens*). These findings confirm the central role of geographical variation in ecology and evolution of plant-herbivore interactions.

The activity of the natural enemies attracted by the EFNs also varied geographically and seasonally in *C. pallida*. Many studies in different systems have demonstrated geographic variation in EFNs defence^27,31,60–63^. In general, the activity of the natural enemies was higher in the periods Jan/Feb and Mar/Apr than May/Jun. This difference among periods may be the result of the amount of nectar offered or the number of active EFNs^34,62,64^. Ants removed more baits than wasps, as in many previous studies of interactions mediated by EFNs^63–65^. However, in the population of Village, when the wasps were present there was an additive effect in the percentage of removed baits. This result contrasts with those obtained by Cuautle & Rico-Gray^25^ which did not observe additive effect of ants and wasps acting together. These findings suggest that, in addition to ant, predatory wasps can play important role in the interaction between *C. pallida* and its herbivores^25,26^.

The ant community composition varied geographically. The populations (Iperó and Martinho Prado) that showed the most percentage of removed baits were visited by ants of smaller body size (*Solenopsis* and *Pheidole*). In contrast, the Village population that had a lower percentage of removed baits was visited by ants of large body size. Sendoya & Oliveira^27^ found a negative relation between the infestation of plants by caterpillars and the presence of ants, and this relation varied geographically depending on the abundance and composition of the ant species. The species composition can be decisive for the plant indirect defence, because characteristics such as body size and ant aggressiveness are critical components for the level of plant benefit^65^. Some ant species are highly aggressive, while others do not act as defenders of plant (e.g. *Cephalotes*)^24,66^. The high percentage of removed baits in the Iperó and Martinho Prado populations may be explained by the aggressive behaviour of the species that were found in these localities^65^. Smaller ant species exhibit worker recruitment behaviour and this may lead to increased plant protection effectiveness^31,34,65^. Nogueira *et al*.^31^ verified in a study with *Anemopaegma album* (Bignoniaceae) that the greatest difference between the outcome of the interactions among the populations was due to differences in the species and behaviour of the ants. Godoy & Camargo^67^ reported that body size influences foraging behaviour, where small ants are not only more abundant and active than large ants, but also recruit more consistently.

Variation in phenotypes in the wild can be the result of genetic variation among plant populations and variation in the environmental factors among sites. To test if part of the large variation in the traits among populations that we observed in the field has a genetic component, we performed a common garden experiment. For the PA concentration on unripe seeds, there was a clear genetic difference among the populations tested. This result confirms other studies on genetic variation of PAs^32,68^. For the attraction of natural enemies to the EFNs, there were no genetic differences among populations, suggesting that all the large variation observed in the original field populations can be caused by the environmental factors discussed above, such as the composition of the ant and wasp communities. In a study with *Alliaria petiolata* (Brassicaceae), Cipollini also found that environmental factors were the main determinant of variation in a chemical defence across natural populations^69^. For the percent of attacked pods by the specialist and the non-specialist herbivore, we did not find a significant effect of population, indicating that most of the variation observed in the original field populations was caused by environmental variation. However, we observed a significant statistical interaction between population and herbivore type suggesting some genetic differentiation among populations in traits affecting attack by the different herbivores.

We expected that the specialist *U. ornatrix* would act as selection agent that decreases the concentration of PAs in natural populations, while the non-specialist *E. zinckenella* would act as a selection agent that increases the concentration of PAs. This expectation is based on the fact that PAs are known to deter generalist herbivores^16,17,70,71^, but have no negative effect on the specialist *U. ornatrix*^36^. *U. ornatrix* can sequester PAs and use them as defences against predators as well as a male pheromone^15,19,35,48,70,72^. Additionally, *U. ornatrix* larvae choose diets with higher concentrations of PAs^37^. Therefore, we tested if the variation in herbivory rates across the 20 populations was correlated with PA concentration. However, we did not find a correlation between concentration of PAs and herbivory rates by the specialist and the non-specialist.

There are several possible hypotheses that can explain this lack of correlation. First, the seasonal variation observed in the abundance of the two herbivores may represent a fluctuating risk of attacks constraining the optimization in the level of defence by the plant. Second, the plant may rely on different types of defences to deter the different herbivores, for example the presence of the EFNs may affect *U. ornatrix* larvae^33,46^ but have no effect on *E. zinckenella* larvae that feed inside a single pod for the entire development^54^. Third, we do not know if the levels of PAs present in the field affect *E. zinckenella*. Previous studies showed that the negative effect of PAs on generalist herbivores depends on the concentration^70,71^. Forth, since *C. pallida* is an introduced species, it has not coevolved with these two herbivores for a long evolutionary time, and therefore may not be well adapted to avoid them^40^. Fifth, other plant defences not measured in this study may be affecting the rates of herbivory. For example, *Crotalaria* plants are known to also have non-proteic amionoacids^73^, lectins^74^ and proteinase inhibitors^75^. Future studies on this system can address these hypotheses.

In conclusion, we showed the importance of studying geographical and seasonal variation, as well as the importance of considering both a specialist and a non-specialist herbivore, to understand a plant-herbivore interaction. We showed that attack by the specialist and the non-specialist herbivore, as well as a chemical defence (PAs) and a biotic defence (EFNs), vary greatly in time and space. We showed that there is genetic variation among populations in herbivore resistance and the chemical defence, but no genetic variation in the biotic defence. We also showed great variation in attack rates by the specialist and the non-specialist herbivore as well as the chemical defence in a very large geographical scale including 20 populations. However, herbivory by the specialist and the non-specialist was not correlated with PA concentration. Our study shows that to understand the evolution of ecological interactions it is crucial to investigate how the outcome of the interaction and the important species traits vary geographically and seasonally^1^.

## Supplementary information

Supplementary Material

Dataset 1

## Data Availability

All original data is provided in the supplemental material.
