# Supplementary material for: The geographical and seasonal mosaic in a plant-herbivore interaction: patterns of defences and herbivory by a specialist and a non-specialist
Source: Sci Rep. 2019 Oct 23;9:15206. doi: 10.1038/s41598-019-51528-8 (PMC6811555; doi:10.1038/s41598-019-51528-8)
Supplement: Supplementary file 1 — Supplementary Material [file 41598_2019_51528_MOESM1_ESM.docx]

**SUPPLEMENTARY MATERIAL**

**The geographical and seasonal mosaic in a plant-herbivore interaction: patterns of defences and herbivory by a specialist and a non-specialist**

Diomar Verçosa^1,3^, Rodrigo Cogni^4,*^, Marcos Nopper Alves^2^, José Roberto Trigo^3, **^

1. Postgraduate Program in Plant Biology, Institute of Biology, University of Campinas - UNICAMP, Campinas-SP, Brazil

2. Laboratory of Plant Tissue Culture, Department of Agrotechnology, Center for Biological and Agricultural Chemistry, UNICAMP, Campinas-SP, Brazil

3. Laboratory of Chemical Ecology, Department of Animal Biology, Institute of Biology, University of Campinas - UNICAMP, 13083-862 Campinas-SP, Brazil

4. Department of Ecology, University of São Paulo, São Paulo 05508-900, Brazil.

*correspondence author. E-mail: [rcogni@usp.br](mailto:rcogni@usp.br)

***in memoriam* Prof. Trigo passed away in November 2017

**Statistical analyses**

To verify if there was geographic and temporal variation in the percentage of attacked pods by herbivore type, we compared among the three populations and among the periods: Jan/Feb, Mar/Apr and May/Jun. The periods when some populations had no unripe pods were not included in the analyses. We used a generalized linear model, with Poisson distribution, with log function link, log-likelihood test of type 1 and deviation correction coefficient to correct overdispersion (McCullagh & Nelder 1989). We compared the concentration of PAs in the unripe seeds, in the three populations, in three different periods. We made this comparison using a two-way ANOVA, where the independent factors were population and period. We transformed the concentration of PAs in ln to meet ANOVA normality assumptions.

To verify if there was geographic and temporal variation in the number of simulated herbivores preyed by ants and wasps, we compared among the three populations and among the periods: Jan, Mar and May. We used a generalized linear mixed model, with binomial distribution.

In the common garden experiment, we compared the height of the plants of the three populations, in each month, using a one-way ANOVA. We compared the percentage of attacked pods by type of herbivore among the three populations in January and April as described above. We compared the PAs concentration (transformed ln) in unripe seeds between the three populations, in each month, using a one-way ANOVA, with a post hoc Tukey’s multiple comparison. The percentage of removed baits among the populations and treatments, in January, were analysed using a generalized linear model, using Poisson distribution, with log function link, log-likelihood test of type 1 and deviation correction coefficient to correct overdispersion (McCullagh & Nelder 1989). We did not incorporate the comparison between the months in the same analysis model of all variables measured in this common garden experiment, because we lost many plants in the field, which died from January to April (Iperó: 78%, Martinho Prado: 35% e Village: 38%), thus, the comparison between the months would be unbalanced. For this same reason, a bait removal bioassay was performed only in January.

To verify if there was geographic and temporal variation in the percentage of attacked pods by herbivore type in a very large geographic scale, we compared between the twenty populations and among the periods: January and May. We used a generalized linear model, with Poisson distribution, with log function link, log-likelihood test of type 1 and deviation correction coefficient to correct overdispersion (McCullagh & Nelder 1989). We compared the concentration of PAs using a two-way ANOVA, where the independent factors were population and period. We transformed the percentage of attacked pods and the concentration of PAs in ln to meet ANOVA normality assumptions.

We tested the hypothesis that populations with higher incidence of the specialist *U. ornatrix* would present a lower PAs concentration, where those with a higher relative incidence of the non-specialist *E. zinckenella* would present a higher concentration of these alkaloids. In populations where both herbivores had a similar incidence, we would expect an intermediate concentration. We used the non-parametric correlation of Spearman with the untransformed data to verify if the mean concentration of PAs correlated with the average percentage of attacked pods by herbivore type in each of the 20 populations in January and 16 in May. In addition, we verified the amplitude of variation of the percentage of attacked pods by herbivore type, as well as the PAs concentration in January and May among the 20 populations.

All analyses were performed with the Statistic program 7.0, StatSoft, Inc. 2004, except the GLMM that were run in the R environment version 3.2.4 using the package lme4.

**Supplementary Figures**


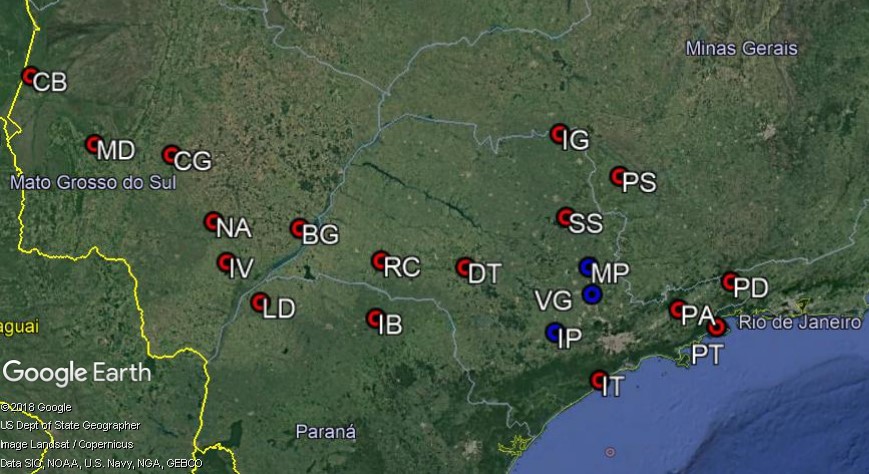


Figure S1. Distribution of the twenty *Crotalaria pallida* populations sampled in two periods (January and May) of 2014 in the states of Paraná, Mato Grosso do Sul, São Paulo and Rio de Janeiro. The blue dots represent the three populations that were sampled initially over a year; later these populations were also sampled in two periods. Map generated in the software Google Earth (7.1.3.29.1) accessed on https://www.google.com.br/earth/


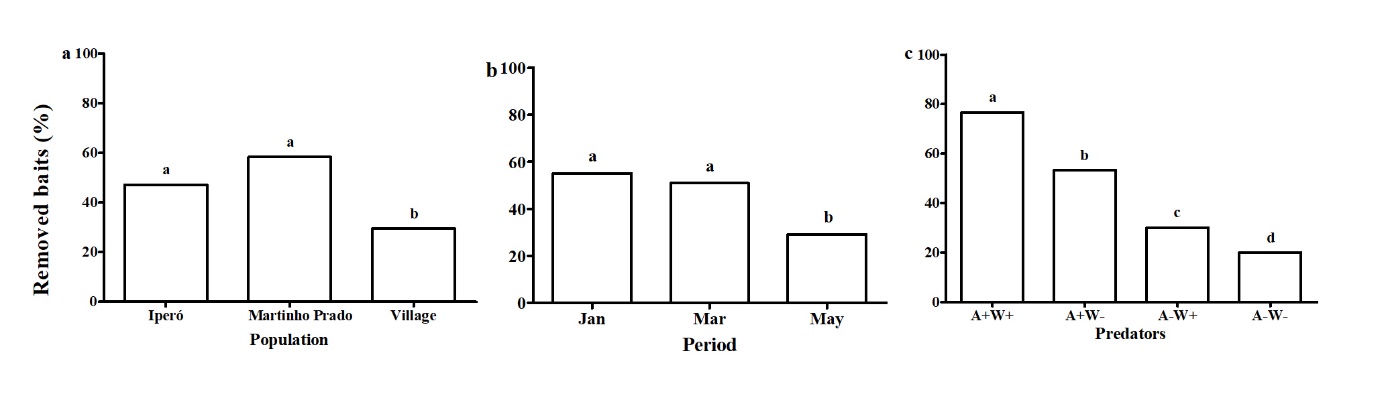


**Figure S2**. Percentage of removed baits in three populations of *Crotalaria pallida* (**a**), in three periods (**b**) and in four treatments (A+W+: with ants and wasps, A+W-: with ants and without wasps, A-W+:without ants and with wasps, and A-W-: without both the predators) (**c**) on experiment at the place of origin. Different letters above each bar represent significant differences (P < 0.05).


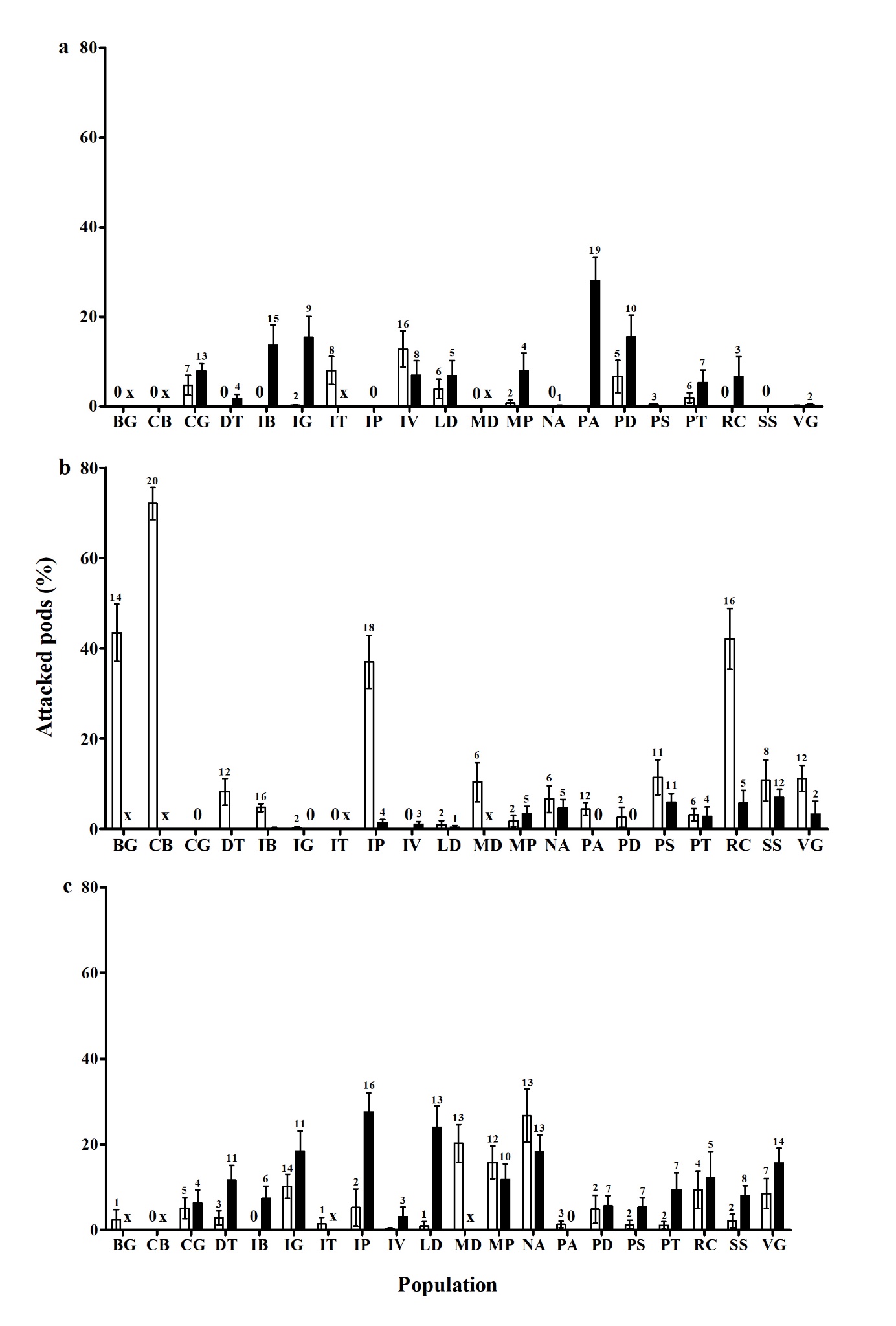


**Figure S3.** Percentage of attacked pods by *Utetheisa ornatrix* (a) and *Etiella zinckenella* (b) or both herbivores (c) in the twenty populations of *Crotalaria pallida* in January (white) and May (black) of 2014. “0” indicates that there was no herbivory on the plants in the population and period. “x” indicates that plants were not found in the in the population in May. Data are present as mean ± standard error. Values above bars indicate the number of sampled plants.


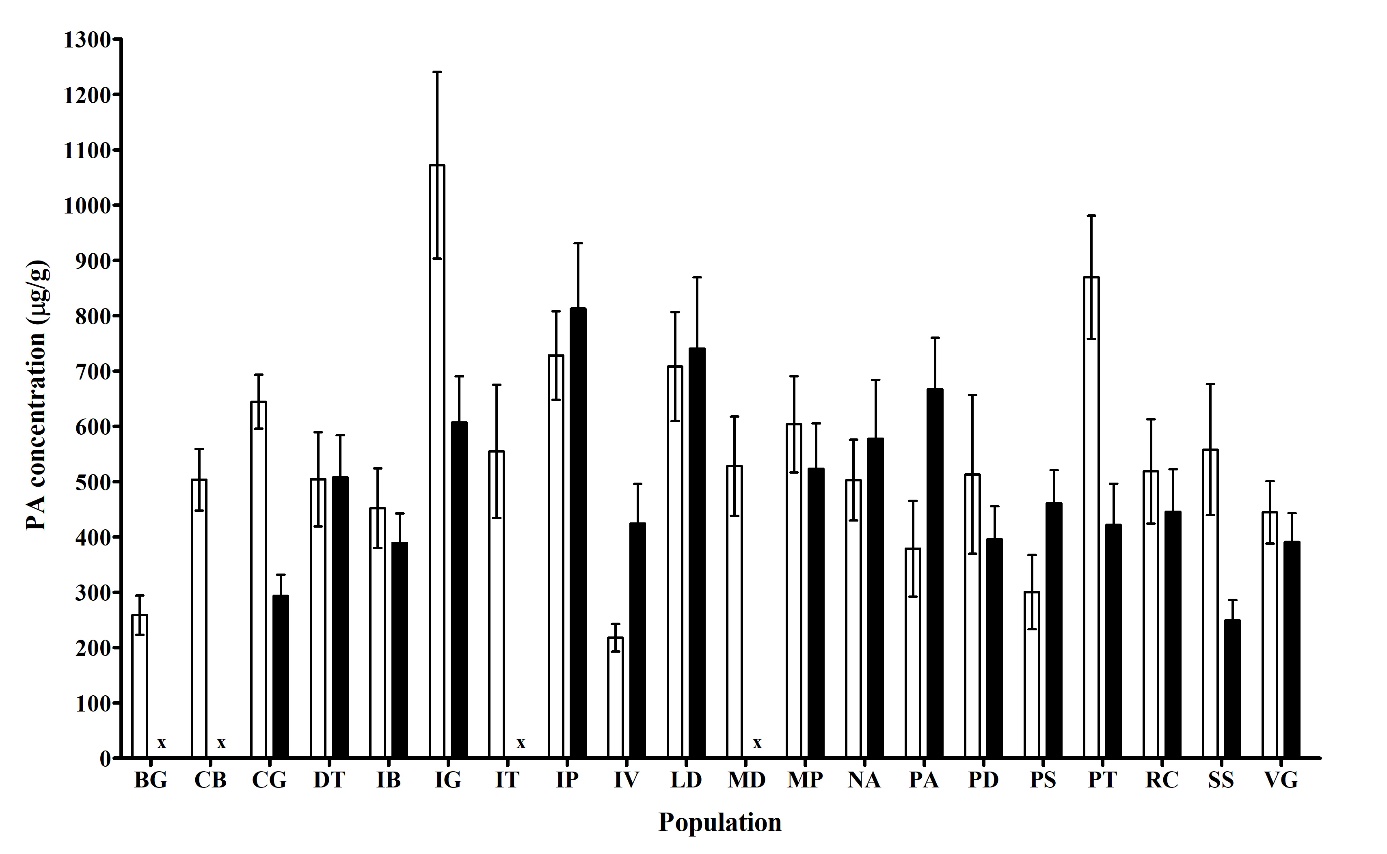


**Figure S4.** Pyrrolizidine alkaloids concentration in unripe seeds in twenty populations of *Crotalaria pallida* in January (white) and May (black) of 2014. “x” indicates that plants were not found in the population in May. Data are present as mean ± standard error.

**Supplementary Tables**

Table S1. Location of the twenty populations of *Crotalaria pallida* sampled in January and May of 2014 in the states of Paraná, Mato Grosso do Sul, São Paulo and Rio de Janeiro.

| Locality, State | Abbreviation | Latitude | Longitude |
| --- | --- | --- | --- |
| Bataguassu, MS | BG | 21º43'50,49"S | 52º25'58,24"W |
| Campo Grande, MS | CG | 20º28'9,64"S | 54º50'25,46"W |
| Corumbá, MS | CB | 19º1'30,23"S | 57º37'6,80"W |
| Duartina, SP | DT | 22º20'50,48"S | 49º22'51,92"W |
| Ibiporã, PR | IB | 23º14'41,40"S | 51º1'16,19"W |
| Igarapava, SP | IG | 20º3'34,27"S | 47º44'24,34"W |
| Iperó, SP | IP | 23º24'43,93"S | 47º42'34,95"W |
| Itanhaém, SP | IT | 24º12'25,69"S | 46º51'22,87"W |
| Ivinhema, MS | IV | 22º18'49,44"S | 53º49'2,24"W |
| Loanda, PR | LD | 22º59'55,92"S | 53º11'33,25"W |
| Martinho Prado, SP | MP | 22º17'7,17"S | 47º8'21,36"W |
| Miranda, MS | MD | 20º15'27,94"S | 56º20'50,17"W |
| Nova Alvorada do Sul, MS | NA | 21º37'7,31"S | 54º3'16,13"W |
| Paraty, RJ | PT | 23º13'28,43"S | 44º44'0,57"W |
| Passos, MG | PS | 20º44'19,28"S | 46º38'25,47"W |
| Penedo, RJ | PD | 22º26'38,05"S | 44º30'54,62"W |
| Pindamonhangaba, SP | PA | 22º56'56,11"S | 45º27'18,60"W |
| Rancharia, SP | RC | 22º15'51,78"S | 50º55'32,97"W |
| São Simão, SP | SS | 21º27'28,76"S | 47º34'37,59"W |
| Village, Campinas, SP | VG | 22º44'41,22"S | 47º3'42,29"W |

Table S2. Results of the GLM for the percentage of attacked pods by herbivore type in three populations of *Crotalaria pallida* on period Jan/Feb, Mar/Apr and May/Jun.

| Effect | df | Log**-**likelihood | χ^2^ | P |
| --- | --- | --- | --- | --- |
| Population | 2 | -4049.84 | 6.727 | 0.035 |
| Period | 2 | -4047.59 | 4.509 | 0.105 |
| Herbivore type | 2 | -3996.40 | 102.376 | < 0.001 |
| Population x Period | 4 | -3981.50 | 29.797 | < 0.001 |
| Population x Herbivore type | 4 | -3967.87 | 27.276 | < 0.001 |
| Period x Herbivore type | 4 | -3950.94 | 33.843 | < 0.001 |
| Population x Period x Herbivore type | 8 | -3930.41 | 41.063 | < 0.001 |

Table S3. Results of the GLMM for the percentage of removed baits by different predators in three populations of *Crotalaria pallida* on period Jan, Mar and May.

| Effect | df | χ^2^ | P |
| --- | --- | --- | --- |
| Period | 14 | 24.06 | 0.045 |
| Population | 14 | 25.05 | 0.034 |
| Treatment | 17 | 58.39 | <0.0001 |
| Period x Population | 6 | 10.33 | 0.111 |
| Period x Treatment | 8 | 5.76 | 0.674 |
| Population x Treatment | 8 | 12.09 | 0.147 |
| Period x Population x Treatment | 12 | 0.47 | 1.000 |

Table S4. Frequency of individual plants visited by each ant visitor of extrafloral nectaries in three populations of *Crotalaria pallida*, on period Jan/Feb, Mar/Apr and Mai/Jun (Sample number = 30).

| Genus | Iperó | Martinho Prado | Village |
| --- | --- | --- | --- |
| *Solenopsis spp* | 18 | 0 | 0 |
| *Camponotus spp* | 8 | 0 | 4 |
| *Pheidole* | 0 | 7 | 0 |
| *Ectatomma* | 0 | 0 | 2 |
| *Pseudomyrmex* | 0 | 0 | 1 |
| *Dorymyrmex* | 1 | 0 | 0 |

Table S5. Results of GLM for the percentage of attacked pods by herbivores in the three populations of *Crotalaria pallida* in January and April on a garden common experiment.

| (A) January |  |  |  |  |
| --- | --- | --- | --- | --- |
| Effect | df | Log**-**likelihood | χ^2^ | P |
| Population | 2 | -4335.20 | 0.390 | 0.823 |
| Herbivore type | 1 | -4333.47 | 3.450 | 0.063 |
| Population x Herbivore type | 2 | -4324.57 | 17.810 | < 0.001 |
| (B) April |  |  |  |  |
| Population | 2 | -2988.92 | 5.470 | 0.065 |
| Herbivore type | 1 | -2984.43 | 8.990 | 0.003 |
| Population x Herbivore type | 1 | -2984.41 | 0.032 | 0.858 |

Table S6. Results of the GLM for the percentage of attacked pods by herbivore type in 20 populations of *Crotalaria pallida* on the periods January and May.

| Effect | df | Log**-**likelihood | χ^2^ | P |
| --- | --- | --- | --- | --- |
| Population | 19 | -37046.7 | 355.822 | < 0.0001 |
| Period | 1 | -36868.7 | 12.133 | < 0.0001 |
| Herbivore type | 2 | -36862.7 | 98.332 | < 0.0001 |
| Population x Period | 15 | -36813.5 | 124.763 | < 0.0001 |
| Population x Herbivore type | 38 | -36751.1 | 1069.812 | < 0.0001 |
| Period x Herbivore type | 2 | -36216.2 | 180.353 | < 0.0001 |
| Population x Period x Herbivore type | 31 | -36126.1 | 160.741 | < 0.0001 |
